# Application of mesenchymal stem cell exosomes and their drug‐loading systems in acute liver failure

**DOI:** 10.1111/jcmm.15290

**Published:** 2020-06-03

**Authors:** Shuqin Zhang, Yu Hou, Jing Yang, Denghui Xie, Linrui Jiang, Huazhong Hu, Jingjing Hu, Caizhu Luo, Qun Zhang

**Affiliations:** ^1^ Office of Clinical Trial of Drug Guangdong Provincial Key Laboratory of Bone and Joint Degeneration Diseases The Third Affiliated Hospital of Southern Medical University Guangzhou China; ^2^ Guangdong Provincial Key Laboratory of Bone and Joint Degeneration Diseases The Third Affiliated Hospital of Southern Medical University Guangzhou China

**Keywords:** acute liver failure, drug‐loading systems, exosomes, mesenchymal stem cell, pre‐clinical application

## Abstract

Stem cell exosomes are nanoscale membrane vesicles released from stem cells of various origins that can regulate signal transduction pathways between liver cells, and their functions in intercellular communication have been recognized. Due to their natural substance transport properties and excellent biocompatibility, exosomes can also be used as drug carriers to release a variety of substances, which has great prospects in the treatment of critical and incurable diseases. Different types of stem cell exosomes have been used to study liver diseases. Due to current difficulties in the treatment of acute liver failure (ALF), this review will outline the potential of stem cell exosomes for ALF treatment. Specifically, we reviewed the pathogenesis of acute liver failure and the latest progress in the use of stem cell exosomes in the treatment of ALF, including the role of exosomes in inhibiting the ALF inflammatory response and regulating signal transduction pathways, the advantages of stem cell exosomes and their use as a drug‐loading system, and their pre‐clinical application in the treatment of ALF. Finally, the clinical research status of stem cell therapy for ALF and the current challenges of exosome clinical transformation are summarized.

## Graphical abstract

Mesenchymal stem cell‐derived exosomes from different sources and their use as drug‐loading systems to repair acute liver failure induced by inflammation, autophagy, apoptosis, various cytokines and signaling pathways,etc.

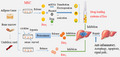

## 1 BACKGROUND

Acute liver failure (ALF) is a rare but lethal clinical syndrome that is defined as the acute development of jaundice, synthetic failure and hepatic encephalopathy in patients without a previous history of liver disease. This is a serious and complicated disease, and the mortality rate is as high as 60% to 90%.^1^ Drug toxicity, viral hepatitis and uncertain factors may be the main causes of ALF. The sudden loss of liver function caused by acute and massive liver cell destruction^2^ further develops into brain oedema, septicaemia and multiple organ failure, which eventually leads to death.^3^ At present, ALF treatment strategies are mainly to reduce disease progression and prevent the occurrence of complications. Clinically, orthotopic liver transplantation, artificial liver therapy and plasma exchange are currently considered the main treatments for ALF patients. However, the lack of donor livers, the short‐term efficacy of artificial liver therapy, the high incidence of post‐operative complications and unaffordable economic costs limit the clinical application of ALF treatments.^4^ Therefore, non‐surgical strategies to increase the spontaneous recovery rate of damaged livers are particularly important. A growing number of studies have shown in recent years that stem cells and their derived exosomes can effectively treat ALF.

Exosomes range in size from 30 to 150 nm,^5^ have a density of approximately 1.13‐1.19 g/mL^6^ and can be transmitted to other cells to perform their functions. Exosomes not only target the target cells to trigger downstream signals but also transfer genetic material to target cells, exerting anti‐inflammatory, antiapoptotic and immunosuppressive effects, promoting tissue repair and improving cytokine levels.^7^, ^8^ Although some breakthroughs have been made in the field of exosomes, the specific biological role of exosomes has not been fully elucidated. We summarized the induced aetiology and pathogenesis of acute liver failure, as well as the therapeutic mechanism and pre‐clinical application of mesenchymal stem cell exosomes in acute liver failure.

## 2 THE ROLE OF EXOSOMES IN THE IMMUNE REGULATION OF ACUTE LIVER FAILURE

### 2.1 Sources of stem cell exosomes

Several types of stem cells, including hepatic progenitor cells (HPCs), mesenchymal stem cells (MSCs) and induced pluripotent stem cells (iPSCs), have been used in the study of liver diseases^9^; among them, MSCs are the most widely used because they have multiple differentiation and self‐renewal potential, are easily accessible and lack ethical issues.^10^ According to the guiding principles of the International Association of Cell Therapy, MSCs must express CD105, CD90 and CD73 and lack expression of CD45, CD34, CD14 or CD11b, CD79α or CD19, and HLA‐DR surface molecules.^11^ MSCs have great therapeutic potential, such as in tissue repair and immune regulation and as promising strategies for the treatment of liver diseases.^12^ However, the exact mechanism by which MSCs play a therapeutic role in ALF is unclear. Studies have shown that because of the low rate of MSC implantation, only a few MSCs can colonize in the liver, and so, the intervention effects of MSCs on diseases are extremely limited, and they are more likely to mediate their therapeutic effects on damaged organs through paracrine mechanisms.^13^ There is growing evidence that exosomes secreted by MSCs are smaller, simpler and less immunogenic than their mother cells and avoid the risk of cell rejection, iatrogenic tumour formation and pulmonary embolism that are associated with MSC transplantation, which is generally superior to the corresponding MSCs.^14^

As early as the 1960s, vesicles were detected in cartilage and suspected to have physiological functions in bone mineralization.^15^ Trams and colleagues first demonstrated that vesicles play a role in cell communication in 1981,^16^ and the term ‘exosomes’ was coined by Johnstone et al in 1987.^17^ However, the standardized criteria for classifying vesicular subtypes are still in the development stage. It is generally believed that vesicles can be divided into three categories: exosomes, microvesicles and apoptotic bodies. These subsets are different in biogenic pathways, size, membrane markers and contents. Exosomes originate from the endosome system and are membranous vesicle nanoparticles coated with a large number of bioactive factors.^18^ According to the proposal of the International Society for Extracellular Vesicles (ISEV) in 2018, exosome identification methods include transmission electron microscopy (TEM), nanoparticle size analysis and Western blotting. With the deepening of research and the updating of technical methods, a wealth of contents of MSC‐derived exosomes has been identified, which mainly mediate the continuous transfer of nucleic acids and proteins through different ways to change the activities of target cells and achieve disease treatment.^7^

### 2.2 Acute liver failure and inflammation

ALF is a process of hepatocyte injury that is dominated by inflammatory reactions, and a variety of hepatotoxic factors, such as concanavalin A (ConA),^19^ acetaminophen (APAP)^19^ and lipopolysaccharide (LPS),^20^ induce immune dysfunction and lead to ALF. Imbalance of the immune response plays a crucial role in the pathological process of ALF.^21^ It is well known that the liver has many important functions in vivo, and one of the main functions is related to innate immunity. Pattern recognition receptors (PRRs) are important molecules in the innate immune system. They recognize pathogen‐associated molecular patterns (PAMPs) that are highly conserved on the surface of pathogenic microorganisms and damage‐associated molecular patterns (DAMPs) that are released by damaged tissues and cells, activate downstream signal transduction pathways, induce inflammatory cell infiltration at injury sites, promote the release of inflammatory factors, further aggravate the inflammatory injury status at injured tissues, and drive and regulate the adaptive immune response.^22^ Among them, infectious inflammatory responses are usually accompanied by the presence or invasion of pathogenic microorganisms, such as bacterial lipopolysaccharide or flagellin, which are mediated by PPR recognition of pathogen‐associated molecular patterns, resulting in complement deficiency or impaired functions of innate immune cells (including monocytes, macrophages, dendritic cells, NK cells and neutrophils), which exacerbates the severity of liver disease. Furthermore, the environment of ALF inflammation and necrosis makes patients prone to systemic infections and leads to multiple organ failure.^23^, ^24^

Contrary to septicaemia or inflammation caused by bacterial and systemic reactions, an increasing number of studies have defined inflammatory responses induced by damage that is mediated by non‐infectious sources. Aseptic inflammation is a common result of many clinical liver diseases. In the absence of any infection, the release of intracellular molecules is driven by DAMPs in solid organs because pathological cell death is the main reason for aseptic inflammation.^25^ Cell membranes become permeable to intracellular contents after cell death.^26^ When cells break down, the contents of the cells or DAMPs are released during complete necrosis, and then, these molecules bind to pattern recognition receptors such as Toll‐like receptors (TLRs), which induce production of inflammatory cytokines (such as TNF‐α, IL‐1β, IL‐6 and IL‐8) and activation of the inflammatory cascade.^27^, ^28^, ^29^ Among them, TNF‐α plays a key role in inflammatory responses during acute or chronic hepatitis caused by viral infection, steatosis, autoimmunity, and alcohol or paracetamol consumption.^30^ IL‐1β is also related to the early inflammatory response, and toxic substances can induce an increase in IL‐1β in the liver. Elevated IL‐1β levels activate immune cells to synthesize and release many inflammatory cytokines that are involved in various immune responses, leading to inflammation and tissue destruction.^31^ IL‐6, an important inflammatory cytokine in animal livers, stimulates the proliferation, differentiation and functional enhancement of cells that are involved in the immune response.^32^ The level of IL‐8 is an index of the severity of the inflammatory response in organs to poison in vivo.^33^ Exposure to toxins first activates TNF‐α and IL‐1β, and induction of TNF‐α and IL‐1β promotes the up‐regulation of pro‐inflammatory cytokines such as IL‐6 and IL‐8 and induces the cascade of pro‐inflammatory cytokines to exacerbate ALF. Therefore, effective treatment strategies to inhibit DAMP‐ or PAMP‐induced inflammatory responses in ALF are urgently needed.

### 2.3 Mesenchymal stem cell exosomes regulate the inflammatory response in ALF

The imbalance of the liver immune microenvironment is important in the pathogenesis of ALF. Further work is needed to change the levels of pro‐inflammatory and anti‐inflammatory cytokines in vivo to reduce uncontrollable organ damage.^34^ In recent years, with the development of regenerative medicine and stem cell technology, stem cell‐based treatment strategies have brought new hope to patients with ALF. To prevent the immune response in the pathogenesis of ALF, MSCs effectively inhibit the levels of the inflammatory cytokines TNF‐α, IL‐1β, IL‐6 and IFN‐γ, and high mobility group box 1 protein (HMGB1) and chemokines (CXCL1 and CXCL2) in the serum and liver through a paracrine mechanism and up‐regulate the expression of TGF‐β, PGE2, IL‐10, SOD, GSH and HGF in ALF models.^35^, ^36^, ^37^, ^38^, ^39^ Further research reports that MSC‐conditioned medium (MSC‐CM, a combination of soluble factors and exosomes) has a similar therapeutic effect in treating liver failure as MSCs. Compared with the control group, MSC‐CM treatment significantly reduced serum IFN‐γ, IL‐1β and IL‐6 levels and increased serum IL‐10 levels.^40^ Forty‐eight hours after MSC‐CM injection, the biochemical and histopathological parameters of mice with acute liver failure were improved, although the survival rate did not increase.^41^ In addition, MSC‐CM has a robust inhibitory effect on fibrogenesis and necrotic inflammation in chronic liver injury by promoting liver regeneration and reducing hepatocyte apoptosis.^42^ Although exosomes are the main substance in conditioned medium that plays a therapeutic role in liver failure, the study of extracting exosomes from stem cell culture supernatant for acute liver failure has been continuously reported. The use of human menstrual blood stem cell–derived exosomes (MenSC‐Exos) to treat D‐GalN/LPS injection–induced fulminant hepatic failure (FHF) mouse models reduced circulating TNF‐α, IL‐6 and IL‐1β levels, improved liver function and ultimately reduced mortality in FHF mice.^43^ In addition, in vitro models showed that mesenchymal stem cells induce hepatocytes to transform into progenitor oval cells by secreting exosomes and repair damaged livers by replenishing necrotic hepatocytes during liver regeneration.^44^, ^45^ Next, we focused on the detailed molecular mechanism of stem cell exosomes in ALF.

## 3 EXOSOMES INHIBIT SIGNAL TRANSDUCTION PATHWAYS IN ALF

In the case of liver cell injury, cell stress leads to the activation of death and survival pathways. Exosomes have been widely accepted as carriers that block multiple signal transduction pathways; therefore, great attention has been paid to the relationship between exosomes and ALF to explore whether they can block various signal transduction pathways to prevent liver inflammation, fibrosis and further deterioration of organ failure.^8^, ^46^ The main signal transduction pathways in acute liver failure are discussed in the following sections.

### 3.1 JNK, NF‐κB, STAT 3 and other signalling pathways

Many previous reports have shown that multiple signalling pathways, including mitogen‐activated protein kinase (MAPK), nuclear factor‐kappa B (NF‐κB) and the nucleotide‐binding domain‐like receptor protein 3 (NLRP3) inflammasome, play an important role in regulating the inflammatory response during ALF.^47^ On the one hand, the MAPK family is composed of c‐jun‐N terminal kinase (JNK), p38 kinase and extracellular signal‐regulated kinase, which regulate the transcription of COX‐2, iNOS, TNF‐α, IL‐1β and IL‐6.^48^ JNK is a stress‐ and mitogen‐activated protein kinase that represents a pro‐apoptotic pathway. Even in the case of cell necrosis, the mechanism of apoptosis is activated to some extent, and the imbalance between survival and death pathways may decide the final outcome.^49^ Excessive inflammatory stress induces oxidative stress in cells and subsequent activation of the JNK signalling pathway.^50^ Translocation of activated JNK to the nucleus promotes the transcription and expression of hepatocyte death‐related genes.^51^ In addition, NF‐κB, which contains p50/p65 and IκB protein inhibitors, is very important to host defence and mediates the expression of pro‐inflammatory mediators and cytokines.^52^ It is known that the combined injection of D‐GalN and LPS^53^ is commonly used to establish an ALF animal model. LPS attack induces pro‐inflammatory cytokine secretion in liver crest cells through pattern recognition by Toll‐like receptors (TLRs). Among them, TLR4 is considered to be the main receptor and signal transduction molecule of LPS and plays an important role as an amplifier produced by the inflammatory response and pro‐inflammatory cytokines.^54^ After ligand recognition, signal transduction involving the MAPK and NF‐κB pathways is triggered, leading to the production of pro‐inflammatory cytokines such as TNF‐α and IL‐6.^55^ Peroxisome proliferator‐activated receptor antagonist (PPAR‐γ) is a nuclear hormone receptor and transcription factor that has been demonstrated to inhibit NF‐κB activity.^56^ Activation of PPAR‐γ reduces the onset of hepatic inflammation and septic shock because of the high level of PPAR antigen expression in the liver. In macrophages, STAT3 inhibits the STAT1 signalling pathway and then decreases the production of IL‐12, IL‐27 and IFN‐γ. STAT3 also inhibits the NF‐κB signal transduction pathway,^57^ resulting in a decrease in IL‐6, MCP‐1 and TNF‐α production. Finally, these cytokines target hepatocytes, increase the activation of several signalling pathways, and promote or prevent liver cell injury during hepatitis. The outcome and progression of liver injury are determined by the balance between these signal transduction pathways.^58^ On the other hand, NLRP3 is a polyprotein complex. Once stimulated by activators such as LPS, NLRP3 proteins converge and bind to ASC adapters and then promote the recruitment of caspase‐1 and activation of the mature forms of IL‐1β, releasing IL‐1β and IL‐18 and leading to an inflammatory response.^59^

**FIGURE 1:**
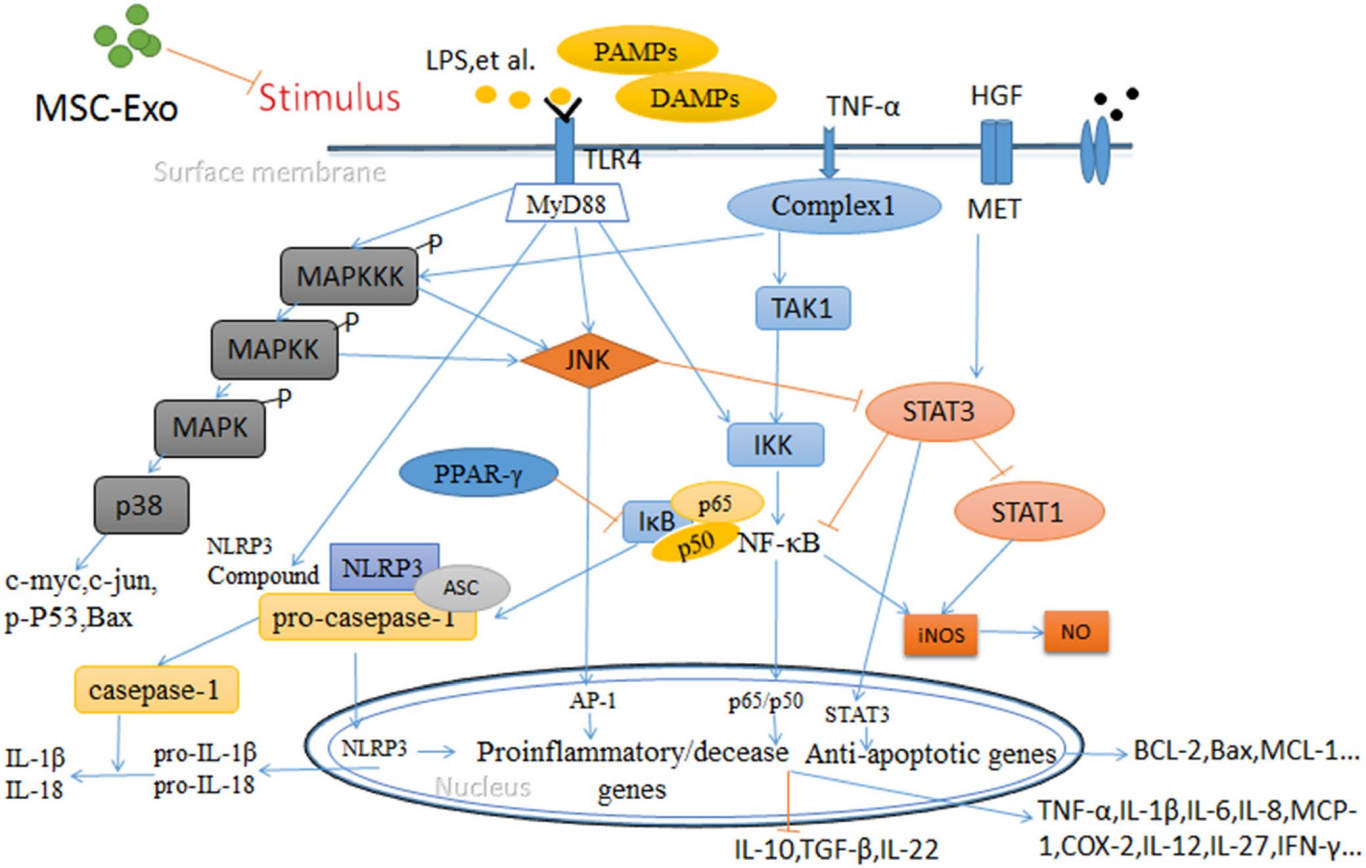
Various factors stimulate activation of relevant pathways to induce acute liver failure, whereas MSC‐Exos alleviate ALF by inhibiting the activation of these signalling pathways. Blue arrow: promote; red arrow: inhibition

Fortunately, studies have shown that exosomes derived from mesenchymal stem cells from different sources inhibit the activation of related signalling pathways in ALF. For example, human endometrial mesenchymal cell–derived exosomes (HuES9.E1 MSC‐Exos) significantly reversed mouse ALF by up‐regulating STAT3 and inhibiting the NF‐κB pathway. CCl_4_‐induced damage in mice can be alleviated by simultaneous administration of HuES9.E1 MSC‐Exos, and the exosome treatment group showed liver gene expression of NF‐κB. Exosomes were subsequently applied to APAP‐ and H_2_O_2_‐treated in vitro models. Compared with the non‐exosome treatment group, exosomes up‐regulated the expression of cell proliferation markers after APAP‐ or H_2_O_2_‐induced injury, demonstrating that MSC‐derived exosomes promote liver cell regeneration and proliferation during acute injury. With exosome treatment, NF‐κB (both p50 and p65) and phospho‐STAT3 activity returned to their normal expression levels before injury. In addition, exosomes also inhibited APAP‐ and H_2_O_2_‐induced hepatocyte expression of apoptotic proteins by up‐regulating Bcl‐xL. However, HuES9.E1 MSC‐Exos did not alleviate liver cell damage by regulating oxidative stress.^60^ To determine the hepatoprotective components in exosomes, a total of 857 proteins in exosomes were analysed using mass spectrometry and antibody arrays.^61^ Among them, IL6ST protein plays an important role in liver cell protection by activating the IL‐6/STAT3 pathway.^62^ Similarly, CXCL2/MIP‐2 restores liver injury by up‐regulating STAT‐3 to enhance liver cell proliferation.^63^ Human umbilical cord mesenchymal stem cell–derived exosomes (hUCMSC‐Exos) significantly reduce serum ALT and AST levels and promote liver cell proliferation by inhibiting macrophage activation.^64^ hUCMSC‐Exos also inhibit oxidative stress‐induced apoptosis by transmitting glutathione peroxidase‐1 (GPX1), up‐regulating ERK1/2 and Bcl‐2 levels, and down‐regulating the IKKB/NF‐kB/Casp‐9/3 pathway.^65^ Exosomes isolated from adipose tissue–derived mesenchymal stem cells (AMSC‐Exos) were administered to treat LPS/GalN‐induced fulminant hepatitis, and marked improvement in liver biochemical indicators was observed. In addition, it was found that AMSC‐Exos are taken up by liver macrophages and reduce the secretion of inflammatory factors (TNF‐α, IFN‐γ, IL‐1β, IL‐6 and IL‐18) in serum and activation of the inflammasome complex (NLRP3, caspase‐1 and ASC) in macrophages. Among them, TXNIP is considered to be a key factor in the activation of NLRP3 inflammasomes, and overexpression of TXNIP reverses the inhibitory effect of AMSC‐Exos on the activation of inflammasomes in LPS‐stimulated macrophages. Further mechanistic studies have shown that AMSC‐Exos contain high levels of miR‐17 family miRNAs, especially miR‐17. AMSC‐Exos protect against ALF through miR‐17–mediated TXNIP inhibitory effects by inhibiting activation of the NLRP3 inflammasome and reducing serum ALT and AST levels. Exosome miR‐17 plays a vital role in the treatment of ALF by targeting TXNIP and regulating inflammasome activation in liver macrophages.^4^ The related mechanism is shown in Figure 1.

### 3.2 The TNF‐α pathway and HGF/c‐Met axis

In addition to the main signalling pathways described above, the TNF‐α pathway also plays a key role in acute inflammatory diseases, including SIRS and ALF, accompanied by acute organ injury. Dysfunctional TNF‐α signal transduction leads to cell death, releases risk‐related molecular patterns and activates tissue‐resident neutrophils and macrophages to secrete TNF‐α and other pro‐inflammatory cytokines/chemokines, which in turn recruit polymorphonuclear leucocytes (PMNs) into damaged tissues and exacerbate cascade reactions. Furthermore, TNF‐α is also the main regulator of tissue repair after acute injury. TNF‐α activates the p38 MAPK and NF‐κB signal transduction pathways, induces cell proliferation and differentiation, activates resident progenitor cells and mobilizes circulating stem cells for tissue regeneration.^66^ The NF‐κB‐ and IL‐6–dependent pathways activated by TNF initiation of liver regeneration involve the STAT3 transcription factor in the G1 phase, whereas damaged hepatocytes that were treated with HuES9.E1 MSC exosomes significantly increased the expression of TNF‐activated NF‐κB and STAT3. The IL‐6/STAT3 pathway subsequently dominates the cell cycle from G1 to S, which is consistent with the up‐regulation of cyclin D1 and PCNA after exosome treatment. All these up‐regulated gene and protein expression levels collectively support the restoration of overall cell viability, suggesting that exosome treatment may mediate liver repair in acute liver injury by inducing liver cell regeneration.^60^, ^67^ In addition, the HGF/c‐Met axis is also related to ALF. The hepatocyte growth factor HGF is the most important mitogen in liver regeneration and repair after liver injury,^68^ and the biological effect of HGF is mediated by a single tyrosine kinase receptor, c‐Met.^69^ The serum level of HGF in liver diseases reflects liver injury and dysfunction,^70^ especially after partial hepatectomy (PH) in rats, and the protein level of HGF and activation of its receptor c‐Met in plasma increased immediately. Overexpression of HGF or application of exogenous HGF induces the proliferation of hepatocytes and accelerates the process of liver regeneration after PH in mice.^71^ When HGF and c‐Met were silenced through RNAi in vivo, liver regeneration was damaged, and the expression patterns of many cell cycle‐ and apoptosis‐related genes were abnormal.^72^ This suggests that HGF/c‐Met signalling is essential for liver regeneration. Proteomic analysis showed that HGF is abundant in MSC‐derived exosomes.^61^ However, further studies are needed to determine whether the HGF/c‐Met signalling pathway is involved in exosome‐mediated hepatocyte dedifferentiation.

### 3.3 Autophagy and apoptosis

In addition to these signal transduction pathways, some recent reports have shown that autophagy is also very important in alleviating the symptoms of specific inflammatory diseases.^73^ Autophagy is a highly conserved intracellular degradation pathway. One of the key mechanisms for the timely removal of damaged mitochondria is a specific form of autophagy called mitochondrial autophagy. Because mitochondrial damage and ATP depletion are the key factors in APAP‐induced liver cell necrosis, it is hypothesized that autophagy may be an important protective mechanism against APAP‐induced liver injury because it helps to remove damaged mitochondria and provide energy for ATP.^74^ There is growing evidence that autophagy is associated with a variety of biological events, including inflammatory responses. Autophagy eliminates damaged organelles or reverses cytoplasmic components by lysosomes in eukaryotic cells and negatively regulates inflammasome activation,^75^ whereas the synergy between exosomes and the autophagolysosomal pathway to reduce intracellular stress conditions is critical in maintaining the balance of intracellular proteins and RNA.^76^ Research has shown that human bone marrow mesenchymal stem cell exosomes (BMSC‐Exos) promote the formation of autophagosomes by increasing the expression of the autophagy marker proteins LC3II and Beclin‐1, and the induction of autophagy effectively reduces liver cell damage after ALF. In particular, the authors found that BMSC‐Exos also attenuate hepatocyte apoptosis by promoting autophagy.^77^

Apoptosis is a process of programmed cell death, which leads to extensive liver injury in acute and chronic liver diseases.^78^ Overwhelming apoptosis constitutes the main pattern of hepatocyte death in some forms of ALF. The antiapoptotic protein (Bcl‐2) family regulates apoptosis in mtDNA‐dependent cells, including Bcl‐2 and the apoptosis‐promoting protein Bax,^79^ both of which regulate downstream caspase‐3 to stimulate protein fragmentation and induce apoptosis.^80^ Apoptosis is usually induced in two ways. One is the death receptor/external pathway, and the other is the mitochondrial/internal pathway. The former is activated by binding of the death signal (such as TNF‐α) with the death receptor on the cell surface. The latter is activated by mitochondrial release of cytochrome c.^81^ These two apoptotic signals can be activated by caspase‐8 and caspase‐9, which further lead to caspase‐3 activation, substrate protein fragmentation, DNA molecular destruction and apoptosis.^82^ Because hepatocytes are rich in mitochondria, which account for 20% of the total oxygen consumption,^83^ it is necessary to study the apoptosis of hepatocytes in ALF. Mitochondria are the downstream targets for inflammatory injury, and oxidative stress is thought to be a trigger that activates a cell apoptosis signal pathway.^84^ Research confirms that prostaglandin (PGE2) secreted by BMSCs attenuates mouse ALF by enhancing hepatocyte proliferation and inhibiting hepatocyte apoptosis.^85^ Furthermore, Zhao S et al showed for the first time that the expression levels of the pro‐apoptotic proteins Bax and caspase‐3 were significantly reduced, and the expression of the antiapoptotic protein Bcl‐2 was up‐regulated after BMSC‐Exos treatment of D‐GalN/LPS‐induced ALF rats. BMSC‐Exos prevent hepatocyte apoptosis through autophagy, thereby inhibiting the occurrence of ALF.^77^ In addition, MenSC‐Exos are absorbed by AML12 cells and migrate to inhibit LPS‐induced AML12 cell apoptosis.^43^ The above studies indicate that MSC‐Exos have the potential to reduce hepatocyte apoptosis after acute liver failure.

## 4 PRE‐CLINICAL STUDIES OF STEM CELL EXOSOMES AND DRUG DELIVERY SYSTEMS IN ALF

With the rise of cell‐free therapy in the field of life sciences, an increasing number of studies have focused on the application of stem cell exosomes themselves and their use as drug carriers in molecular medicine and critical care, including ALF. Compared with traditional synthetic drug delivery systems (DDSs), exosomes or exosomal mimics have many desirable characteristics as ideal drug delivery systems, such as low immunogenicity, the ability to penetrate the blood‐brain barrier,^86^ superior biocompatibility and an absence of inherent toxicity.^87^

### 4.1 Stem cell exosomes and their advantages as drug delivery systems

Exosomes were once thought to be an unnecessary ingredient discarded by cells. However, in the past decade, they have become an important intercellular communication vector for regulating or mediating multiple cell processes.^14^ The most commonly used exosomes are derived from mesenchymal stem cells that are untreated or pre‐treated with hypoxia, serum deprivation and physical stimulation or modified with therapeutic molecules (including miRNAs and cytokines).^14^ Studies have shown that these exosomes can be used in the treatment of a variety of diseases, including central nervous system diseases, myocardial ischaemia/circulation injury, tissue repair and the regulation of tumour metastasis.^88^ In addition, stem cell exosomes are an ideal carrier to escort therapeutic molecules to appropriate targets. By encapsulating the molecule within a membrane, exosomes protect enzymes or RNA from degradation and be taken up by relevant cells through endocytosis.^89^ Because exosomes are nanometre‐sized particles, they can be easily transported through blood and other biological liquids^90^; if this specific exosome‐based transport can be controlled, exosomes may be an effective tool to transfer therapeutic elements (small molecular drugs, viruses, biotherapeutic agents, etc). Because the lipid and surface protein composition of exosomes may affect their function, it is very important to carefully study and consider the biological characteristics of exosomes from different cell types and weigh their benefits and shortcomings for therapeutic purposes. Many teams have studied the use of tumour cell–derived exosomes for the delivery of chemotherapy or other anticancer agents. The advantage is that these exosomes have a specific targeting ability,^91^ but it has been found that related miRNAs and other nucleic acids in tumour‐derived exosomes may lead to malignant changes in target cells.^92^ Some researchers believe that exosomes isolated from fruits and plants are reliable and safe; however, these exosomes do not enhance the function of the host immune system and lack the benefits of immunotherapy.^93^, ^94^ In addition, exosomes derived from immune cells, including monocytes and macrophages, have been shown to be particularly adept at evading immune phagocytosis.^95^ Their role in cancer is well‐defined; however, one potential consequence of their large secretion of exosomes under pathological conditions is the creation of an inflammatory microenvironment that may eventually lead to an increased burden of metastasis.^96^ Among all the known exosome‐generating cell types, MSCs have obvious advantages as a source of exosomes. First, MSCs can be obtained from almost all human tissues and come from a wide range of sources. Second, they can release more exosomes than other cell types. It has been verified that MSC‐derived exosomes have good therapeutic effects and immunosuppressive activity in animal models and show high stability and sustainability in human plasma at −20°C. Furthermore, when cells are immortalized to produce permanent cell lines, the quantity and quality of exosome production are not affected, thus ensuring a sustainable and reproducible supply of exosomes.^97^ Therefore, MSCs are an ideal candidate for the mass production of exosomes for drug delivery.

### 4.2 Types of drugs loaded in stem cell exosomes

The types of exosome‐loaded therapeutic drugs include biomolecules that are difficult to deliver intracellularly without the use of carriers, such as miRNAs, siRNAs and recombinant proteins, as well as synthetic small‐molecule drugs.^98^ Exosome‐loaded small‐molecule drugs are commonly used to target cancers.^99^ Through the fusion of target proteins and exosomal constituent proteins, specific modification of target proteins and other strategies, loading specific proteins into exosomes increases the target protein content in exosomes for immunotherapy.^100^, ^101^ In addition, exosomes can carry nucleic acids to target cells and induce genetic modification during pathogenesis to provide therapeutic genetic material to change gene expression in some diseases and improve gene therapy.^86^ In gene therapy, siRNA is used to destroy genes of interest. However, these siRNAs are unstable and degrade rapidly in systemic circulation. Exosomes can be used as therapeutic vectors to protect and deliver siRNA to target cells.^102^ miRNAs are short non‐coding RNAs that control the post‐transcriptional expression of genes by binding to the complementary sequences of the target mRNA. It is known that exosomes naturally carry miRNAs, and so it is convenient and reasonable to use exosomes as a therapeutic vector to deliver miRNAs to target cells, and this is widely studied.^103^ For example, stem cells were pre‐treated with IL‐1β (βMSC) and used in a sepsis model to observe their effect on liver, lung and kidney organ damage caused by sepsis. Compared with the effects of untreated MSCs, histological evaluation of liver tissue sections in the βMSC‐treated model group showed a significant reduction in oedema, inflammatory cell infiltration and severe bleeding, and serum levels of liver enzymes, AST and ALT were also significantly reduced. Moreover, βMSC‐derived exosomes enhance the immunoregulatory properties of βMSCs in vitro and in vivo. Importantly, the anti‐inflammatory microRNA miR‐146a is strongly up‐regulated by IL‐1β stimulation and selectively packaged in exosomes. The exosomes are then loaded with highly expressed miR‐146a and transferred to macrophages to induce M2 polarization, which regulates their immune function by targeting the IRAK1, TRAF6 and IRF5 signal cascades. Exosome‐loaded miR‐146a plays a vital role in protection against sepsis‐induced organ damage.^104^ In addition, pre‐treatment of human umbilical cord mesenchymal stem cell–derived exosomes (T‐Exos) with TNF‐α has great potential in the treatment of acute liver failure by inhibiting the liver inflammatory response and releasing pro‐inflammatory cytokines from macrophages and can significantly inhibit the recruitment and activation of the NLRP3 inflammasome complex on the trans‐Golgi network (TGN), thereby reducing liver cell degeneration and organ necrosis. T‐Exos play a role in treatment mainly through loading of hsa‐miR‐299‐3p.^105^ When MSCs are stimulated with cytokines, their medium can simulate or improve the biological function of MSCs and transfer the required biological information through exosomes.^106^ Enriching microRNAs or other biologically active substances into exosomes to treat liver injury‐related diseases has become a new idea. However, more in‐depth research is needed to determine the effectiveness of exosome delivery of exogenous siRNA or miRNA, and there is still much room for improvement in using stem cell exosomes as drug delivery systems. The therapeutic potential of exosomes depends on their natural surface membranes and bioengineering capabilities. When designing exosome‐based therapies, it is very important to consider the compatibility of the origin cells and target cells, as well as the stability and appropriate storage conditions of exosomes.^100^

To date, MSCs from different sources have shown good therapeutic effects on ALF^107^ and have entered clinical research, but there are few studies on exosomal modifications and use in drug delivery in acute liver failure that focuses on pre‐clinical applications. Because MSC‐derived exosomes containing therapeutic molecules can be produced in large quantities, the therapeutic role of exosomal‐mediated drug delivery in ALF should attract great attention.

### 4.3 Pre‐clinical study of exosomes and drug delivery systems in treating ALF

Although the medical application of exosomes as biological nanoparticle therapeutic agents is still in its early stages of development,^108^ intravenously injected exosomes preferentially accumulate in the liver and reduce renal clearance, making them particularly suitable for the treatment of liver diseases.^109^ In a mouse model, the plasma half‐life of intravenously administered exosomes is approximately 4 minutes, which is mainly because of liver uptake.^110^ In the case of liver injury, this homing ability is further enhanced^111^ so that exosomes can be rapidly and predominantly distributed in the liver to maximize their therapeutic effect. Because of the many advantages of exosomes in the treatment of liver diseases, exosomes have attracted increasing interest from basic and clinical researchers. MSC‐Exos from various tissues can improve liver function by reducing serum ALT and AST levels and the secretion of inflammatory factors, promoting liver cell proliferation and maintaining liver metabolic homeostasis. Studies have reported that treatment of D‐GalN + LPS‐induced ALF rat models with extracellular vesicles (EVs) derived from human adipose stem cells (hASCs) improved survival rates by more than 70% compared with those of the control groups. Through whole‐genome RNA sequencing and bioinformatic analysis, we showed that human long‐chain non‐coding RNA (lncRNA) H19 increased after EV transplantation. It was further confirmed that lncRNA H19 released by hASC‐EVs is the main mediator of therapeutic effects by promoting the proliferation of liver cells in ALF rats. At the same time, up‐regulation of the HGF/c‐Met pathway was found, suggesting that hASCs‐EVs promote liver cell proliferation through the HGF/c‐Met pathway. Then, we explored the mechanism by which lncRNA H19 affects the HGF/c‐Met pathway and found that hASCs‐EVs promote the proliferation of liver cells by regulating the expression of HGF, STAT3 and PI3K.^112^ In addition, AMSC‐Exos significantly alleviate acute liver failure by inhibiting NLRP3 activation through high expression of miR‐17.^4^ CPMSC‐Exos, which overexpress microRNA‐125b, help liver regeneration by inhibiting activation of Hedgehog (Hh) signalling.^113^ MenSC‐Exos enhance survival and inhibit hepatocyte apoptosis in mice with fulminant hepatic failure by secreting factors such as ICAM‐1, angiopoietin‐2, IGFBP‐6 and osteoprotectin.^43^ Therefore, we believe that MSC‐Exos represent a very attractive treatment. Although different studies have examined the different mechanisms of different stem cell exosomes on liver repair, it is worth noting that these effects are interrelated. The main therapeutic studies of stem cell exosomes as drug delivery systems in ALF are shown in Table 1.

**TABLE 1:** Study on the role of stem cell exosomes from different sources and their loaded biomolecules in animal models related to liver failure

MSC‐Exo type	Loaded small molecule	Animal	Disease model	Function and Ref.
HASC‐Evs	lncRNA H19	Sprague‐Dawley rat	D‐aminogalactose‐induced ALF	Promote hepatocytes proliferation and improve survival rate^112^
AMSC‐Exos	miR‐17	C57BL/6 mouse	LPS/GalN‐induced ALF	Targeting TXNIP and inhibiting the activation of NLRP3 inflammatory bodies in macrophages^4^
MenSC‐Exos	ICAM‐1, osteoprotegerin, angiogenin‐2, etc	C57BL/6 mice	D‐GalN/ LPS‐induced fulminant liver failure	Improves survival and inhibits apoptosis of hepatocytes^43^
hucMSC‐Exos	GPX1	BALB/c‐nu/nu mice	Liver failure caused by CCl_4_ and H_2_O_2_	Reduction in oxidative stress and apoptosis^65^
BMSC‐Exos	miR‐223	C57BL/6 mice	Induction of autoimmune hepatitis by S100	Serum ALT and AST levels were decreased and NLRP3 and caspase‐1 expression was down‐regulated^114^
AMSC‐Exos	miR‐122	C57BL/6 mice	CCl_4_‐induced liver fibrosis	Inhibition of HSC activation and reduction in collagen deposition^115^
CPMSC‐Exos	microRNA‐125b	SD rats	CCl_4_‐induced liver fibrosis	Inhibition of hedgehog (Hh) signal transduction is helpful to liver regeneration^113^
βMSC‐Exos	miR‐146a	C57BL/6 mice	Caecal ligation and puncture‐induced sepsis	Reduce liver damage and improve survival^104^
hucMSC‐Exos	miR‐299‐3p	C57BL/6 mice	LPS+GalN‐induced ALF	Inhibits activation of NLRP3‐related pathways and reduces inflammation^105^

## 5 CLINICAL PROSPECTS AND CHALLENGES

### 5.1 Clinical trials of MSCs in the treatment of liver failure

MSCs have been evaluated in several clinical trials, of which 236 are associated with liver disease (ClinicalTrials.gov), including six for the treatment of acute chronic liver failure. In an open‐label randomized controlled study, umbilical cord mesenchymal stem cell (UC‐MSC) combined with plasma exchange (PE) therapy reduced mortality and adverse outcome rates in 110 patients with hepatitis B virus‐related acute chronic liver failure (HBV‐ACLF), but long‐term efficacy should be further evaluated.^116^ Another phase I‐phase II, open‐label, clinical study evaluated the safety and tolerability of a single infusion of MSCs in liver transplant recipients. Ten liver transplant recipients who received standard immunosuppression received an infusion of 1.5‐3 × 10^6^ MSCs/kg 3 ± 2 days after surgery. Side effects of MSC infusion were not detected on day 3 after liver transplantation, but this infusion did not improve tolerance. This study opens the way for further MSC‐ or Treg‐based trials in liver transplant recipients.^117^ In addition, there is recruitment for a clinical trial on the safety and efficacy of mesenchymal stem cell transplantation for acute chronic liver failure that is expected to be completed on 2022.10.01. In addition to the standard drug treatment (SMT), the experimental group was also treated once a week through peripheral intravenous infusion of 1.0‐10 × 10^5^ MSCs/kg for 4 weeks, and the results have yet to be published (ClinicalTrials.gov, NCT03863002). These studies indicate that the clinical effects of stem cell therapy in patients with ACLF are satisfactory in the short‐term. However, more attention should be focused on large multicentre clinical trials and further basic research. Moreover, well‐designed RCTs are needed to verify the clinical effectiveness of long‐term outcome.^118^ The six clinical trials of MSCs in patients with acute chronic liver failure are shown in Table 2.

**TABLE 2:** Relevant contents and status of clinical trials of MSC in the treatment of patients with liver failure conducted by different research institutions currently searched online

Title	Status	Study results	Conditions	Interventions	Locations
Mesenchymal stem cell transplantation for acute‐on‐chronic liver failure	Recruiting	No results available	Acute‐on‐chronic liver failure	Procedure: mesenchymal stem cell transplantation or placebo infusion via peripheral vein	Changcun Guo, Xi'an, Shaanxi, China
UC‐MSC infusion for HBV‐related acute‐on‐chronic liver failure	Unknown status	No results available	Liver failure	Drug: umbilical cord blood mesenchymal stem cells	/
Safety and efficacy of mesenchymal stem cell transplantation for acute‐on‐chronic liver failure	Not yet recruiting	No results available	Liver failure, acute on chronic	Biological: mesenchymal stem cell	Tianjin Weikai Bioeng. Ltd, Tianjin, China
Mesenchymal stem cells after renal or liver transplantation	Unknown status	No results available	Liver failure/kidney failure	Biological: mesenchymal stem cells	University Hospital Liege, Liege, Belgium
Umbilical cord mesenchymal stem cells transplantation combined with plasma exchange for patients with liver failure	Unknown status	No results available	Liver failure	Other: conventional plus UC‐MSC treatment/other: conventional plus PE treatment/other: conventional plus UC‐MSC and PE therapy/other: conventional treatment	Department of Infectious Diseases, The Third Affiliated Hospital of Sun Yat‐sen University, Guangzhou, Guangdong, China
Intraoperative dialysis in liver transplantation	Terminated	No results available	Liver failure/acute kidney disease/multi‐organ failure	Device: continuous renal replacement therapy (CRRT) Procedure: standard intraoperative support	Division of Critical Care Medicine, University of Alberta Hospital, Edmonton, Alberta, Canada

Although no clinical results of MSC‐Exos in liver diseases have been reported online, there is a recent trend in clinical research using extracellular vesicles obtained from MSCs.^119^ In addition to these published studies, more exosome‐related clinical trials of various autologous or allogeneic sources are ongoing. MSC‐Exos and their application as drug carriers have gradually become promising clinical treatment strategies, including in acute liver failure.

### 5.2 Current challenges

Although most basic studies report that MSC‐derived exosomes are more effective than MSCs in the treatment of complex diseases, because of the lack of appropriate controls, equivalent doses, evaluation of different disease end‐points and diversity of differently originated exosomes, such as pre‐treatment of MSCs (such as serum starvation, hypoxia or inflammation) to change the volume and content of exosomes and stimulating exosome release, it is difficult to draw this exact conclusion, which has led to an increase in the difficulty of clinical treatment. In addition, each laboratory has its own preferred method, and so a standardized programme is needed to analyse and predict exosome content, efficacy and dose to ensure in vivo effects.^120^ As MSCs are heterogeneous and their exosomes are the same, this heterogeneity may have different effects on the target cells they act on.^121^ Improving the delivery efficiency of exosome‐loaded drugs is important for their therapeutic applications.^122^ Therefore, to significantly advance exosome‐mediated therapy in clinical trials, effective methods to maintain the homogeneity of MSC‐derived exosomes, including standardized safety data from pre‐clinical animal models, need to be developed. In summary, more systematic research is needed to clarify the application of exosomes and their drug delivery capacity in complex diseases, including acute liver failure. Therefore, establishing a safer and more effective cell‐free therapy to repair liver damage will reduce the clinical mortality of ALF and improve prognosis in the near future.

## CONFLICT OF INTEREST

The authors confirm that there are no conflicts of interest.

## AUTHOR CONTRIBUTIONS

Shuqin Zhang conceptualized and drafted the manuscript. Yu Hou, JingYang and Denghui Xie assisted with initial drafting and instructive layout of figures and tables. Linrui Jiang, Huazhong Hu, Jingjing Hu and Caizhu Luo gave constructive comments. Qun Zhang critically reviewed and proofread the manuscript and revised the final version. All authors read and approved the final manuscript.

## Data Availability

This is an open‐access article under the terms of the Creative Commons Attribution License, which permits use, distribution and reproduction in any medium, provided the original work is properly cited.
